# Factors associated with rearrest by police among people suspected of an offence and referred for psychiatric assessment: a comparison within a post-booking diversion program in Amsterdam

**DOI:** 10.1186/s12888-026-08143-5

**Published:** 2026-05-13

**Authors:** Jeroen Bastiaan Zoeteman, Hans Joachim de Haas, Louk van der Post, Thimo Martijn van der Pol, Thomas van der Meer, Mathilde Johanna Francina van Oudenaren, Erik Peter Kornelis Sikkens, Jack Dekker, Cornelis Lambertus Mulder

**Affiliations:** 1 Research Department, Arkin Mental Health Institute, Klaprozenweg 111, Amsterdam, The Netherlands; 2 Department Psychiatry, Erasmus MC, Dr. Molewaterplein 40, Rotterdam, The Netherlands; 3 Spoedeisende Psychiatrie Amsterdam, 1e Constantijn Huygensstraat 38, Amsterdam, 1054 BR The Netherlands

**Keywords:** Recidivism, Repeated arrest, Rearrest, Psychiatric disorders, Post-booking diversion program, Homelessness

## Abstract

**Background:**

Mental disorders are highly prevalent among offenders. In Amsterdam, the Psychiatric Emergency Service and the Public Prosecutor’s Office jointly implemented a post-booking diversion program to improve clinical outcomes and reduce recidivism among people suspected of an offence and referred for psychiatric assessment.

**Aim:**

To identify demographic, clinical and legal factors associated with rearrest among participants in a post-booking diversion program.

**Methods:**

In this prospective observational study, 292 participants were followed for one year. Using routinely collected demographic, psychiatric, and judicial data, we applied a two-step negative binomial regression analysis to examine associations between demographic, clinical, and legal variables and the number of rearrests. First, bivariate associations between potential predictors and the number of rearrests were examined. Second, a comprehensive multivariate model was fitted including all significant predictors from step 1.

**Results:**

During follow-up, 42% of participants were rearrested. Participants with substance use disorders initially showed a higher rate of rearrest (Incidence Rate Ratio (IRR) = 1.92, 95% Confidence Interval (CI) 1.17–3.15, *p* = .01), but diagnostic category was no longer associated with rearrest after adjustment for covariates. Independent predictors of rearrest included receiving a subpoena or arraignment (IRR = 2.70, 95% CI 1.48–4.93, *p* = .001), homelessness (IRR = 2.41, 95% CI 1.32–4.37, *p* = .004), welfare dependence (IRR = 1.54, 95% CI 1.01–2.36, *p* = .045), and prior-year arrests (IRR = 1.16, 95% CI 1.07–1.27, *p* < .001).

**Conclusions:**

In this post-booking diversion program, social and legal vulnerabilities—rather than psychiatric diagnosis—were associated with rearrests. Addressing homelessness, economic instability, and prior justice involvement may be more effective in reducing recidivism than interventions focusing solely on mental health treatment. Broader diversion strategies integrating social support and housing in itiatives are warranted.

## Background

The prevalence of serious mental illness in individuals arrested, incarcerated in jails and prisons, or under supervision is between 7 and 18%, generally twice that found in community samples, which is estimated between 3 and 7% [1–5]. Substance use disorders are even more prevalent in justice-involved populations, with international systematic reviews reporting rates ranging from approximately 30% to over 50%, frequently co-occurring with other psychiatric disorders [6]. This disparity has been attributed to several factors. First, closure of large state institutions and psychiatric hospitals in the 1950s and 1960s (known as deinstitutionalization) without a concomitant increase in outpatient capacity, has inadvertently led to the *criminalization* of deviant behavior [7]. While this process unfolded rapidly in the United States, Western Europe—and the Netherlands in particular—followed a more gradual transition away from institutional care, with acceleration only from the mid-1990s [8, 9]. Second, the arrest rather than hospitalization of individuals with serious mental illnesses for minor offences has been partially attributed to law enforcement’s role as first responders, their limited ability to recognize psychiatric conditions (“judicial orientation”), and the lack of access to mental health services [10]. Third, individuals with mental illness who become involved in the criminal justice system frequently experience multiple forms of social vulnerability, such as homelessness, poverty, and unstable living conditions, which have been associated with repeated justice contact [11]. At the same time, established criminogenic risk models emphasize factors such as antisocial attitudes, antisocial peers, and prior criminal behavior as key predictors of recidivism [12]. Fourth, not only may imprisonment itself exacerbate mental health problems and increase the risk of reoffending [13], but also prisoners with major psychiatric disorders are more likely than those without to have had previous incarcerations [14]. While psychosis shows a modest correlation with recurrent crime [15], research shows that primarily substance abuse disorders—with or without concurrent psychiatric illnesses—heightens the risk of rearrest, rather than psychiatric conditions alone [16–18].

Taken together, prior research suggests that criminal justice involvement among people with behavioral health conditions reflects the interplay of psychiatric vulnerability, adverse social circumstances, and system-level responses rather than diagnostic status alone. Given the complexity of factors driving the overrepresentation of behavioral health conditions in the criminal justice system, a comprehensive approach is crucial [19, 20]. As a partial solution, specialized programs have been developed to divert people with mental illness from contact with law enforcement, courts, and corrections to the community, to reduce re-offending and improve mental health outcomes. Diversion from the criminal justice system for persons with mental illness can generally occur at three junctures: pre-arrest diversion, in which police use their discretion not to lay a charge; post-booking or court (pre-arraignment) diversion program, when charges have been laid but diversion into treatment occurs pretrial; and prison-based or post-arraignment diversion, when diversion occurs postplea (e.g. mental health courts) [21, 22].

However, diversion programs also carry the risk of *psychiatrization*: the excessive transfer of persons to psychiatric care based on the assumption that dangerous or criminal behavior results from a psychiatric disorder, without adequate psychiatric examination [23]. This may (partially) explain the finding that prearrest diversion was associated with an increased longer-term risk of rearrest (CIT, Portland) [24, 25]. Post-booking programs have the potential to limit these drawbacks, as they allow for concurrent juridical investigation and psychiatric evaluation before deciding on (a combination of) psychiatric treatment and incarceration. To optimally accomplish this, close collaboration between the prosecutor and psychiatrist is required [26].

Most evidence on diversion and rearrest derives from North American settings. In the Dutch context, characterised by relatively extensive social welfare and healthcare provisions, it remains unclear to what extent rearrest following diversion is associated primarily with psychiatric diagnosis or with markers of social marginalisation such as homelessness and lack of welfare support.

In most municipalities in the Netherlands, all individuals arrested for criminal behavior are taken to the police station, even if they show signs of a mental health crisis [27]. When confronted with a person experiencing a potential mental health crisis, prosecutors commonly decide whether to prosecute or transfer to emergency psychiatric services, without further consultation with a psychiatrist. This fragmented approach often forces a choice between legal action and psychiatric intervention, overlooking the benefits of an integrated strategy. Such misalignment can undermine care and safety, increasing preventable recidivism and the risk of unintended criminalization (or psychiatrization).

In 2017, a specialized post-booking diversion program was launched in Amsterdam, the Netherlands involving close cooperation between the Psychiatric Emergency Service Amsterdam (PESA) and the Public Prosecutor’s Office. One of the purposes of the project was to reduce the risk of repeat contact with the police by identifying behavioral health problems as early as possible in the criminal justice process and diverting them directly into care.

The present study (*N* = 292) aimed to examine demographic, clinical and legal factors associated with rearrest in a Dutch post-booking diversion cohort, with particular attention to the relative contribution of psychiatric diagnoses and social vulnerability.

## Methods

### Terminology

We use person-first language throughout. Our study population comprised people suspected of an offence who were brought into police custody in an acute context. Police may request an urgent psychiatric assessment by the PESA. We refer to a person for whom such an assessment was requested as person suspected of an offence and referred for psychiatric assessment (PSORPA). This term reflects a referral process and does not imply a psychiatric diagnosis.

### Ethical approval

Since participation in this study did not involve any deviation from the established standard care (with the post-booking program considered standard care post-implementation), the project was not subject to the Dutch Medical Research Involving Human Subjects Act (Wet Medisch onderzoek, WMO). Therefore, the study received an exemption from a full review by the IRB (Medical Ethics Review Committee, METC) of Amsterdam University Medical Centre.

### The post-booking program

The new post-booking diversion program entails that when a police officer concludes that a PSORPA is having mental health issues, the police department will directly call the PESA, which in turn aims to visit the PSORPA in the police holding cell within six hours, to conduct the assessment. The assessment is carried out by a psychiatrist or a psychiatry resident indirectly supervised by a psychiatrist. After the assessment, the consulting psychiatrist calls the prosecutor to inform him/her of the presence or absence of the various criteria for involuntary treatment and records this verbal exchange in the medical record. Finally, the prosecutor decides whether to (1) release the PSORPA without further psychiatric treatment, (2) prosecute the patient without further psychiatric treatment, (3) transfer to the PESA department for further psychiatric treatment, or (4) transfer to a prison with a psychiatric ward to allow for a combination of criminal proceedings and psychiatric treatment. Upon transfer to the PESA, the PSORPA undergoes a comprehensive reassessment. This psychiatric evaluation may lead to involuntary admission, voluntary admission, or discharge, with or without referral for further outpatient treatment.

### Study design and recruitment

The conducted study is a prospective observational study with a one-year follow-up using data from a larger cohort study concerning the post-booking diversion program. For this larger study (*N* = 1004), all PSORPAs were enrolled who underwent a psychiatric evaluation as part of the post-booking diversion program during the period of data collection from April 3rd 2017 until August 15th 2020. The initial descriptive study found that 81% of the individuals enrolled in the program had a psychiatric disorder, 39% had a psychotic disorder. Individuals with a psychotic disorder were the ones with the highest likelihood of (involuntary) hospitalization and dismissal of charges [28].

For the present study (*N* = 292), two additional inclusion criteria were applied: (a) residence in the city of Amsterdam, demonstrated either by a registered residential address or, for individuals experiencing homelessness, a designated postal address within Amsterdam; and (b) availability of one‑year follow‑up data. Individuals who were not registered as residing in Amsterdam were excluded because they may have moved out of the city and consequently fallen outside the scope of both justice and mental healthcare services. This could result in missing outcome information and potential false negatives in the follow‑up period. Throughout the manuscript, this sample is therefore referred to as the Amsterdam-based study cohort.

### Data sharing and collection

Demographic data were collected from the Amsterdam municipal department of work, participation and income (WPI). Clinical data were derived from files available within the PESA and the largest mental health care institute of Amsterdam Arkin Mental Health. Legal data were collected from the Amsterdam police department and the Amsterdam public prosecutor’s office. After collection of datafiles within Arkin’s Research Department, they were linked to form one dataset used for analysis. The datafile was pseudonymized before being provided to the researchers. An independent legal consultancy firm was contracted to develop a data-sharing protocol that adhered to the relevant laws and regulations, which was formalized in a covenant signed by all participating parties.

### Measures

With regard to demographics, gender, age, nationality (Dutch, European other than Dutch, and country outside of Europe), having a registered residential address or only a municipal postal address were recorded. In the Dutch administrative system, such postal addresses are commonly assigned to individuals without a fixed residence and were therefore used as an indicator of homelessness. Welfare status was also recorded.

With regard to clinical data, diagnostic classifications were derived from routine emergency psychiatric assessments conducted at the police station. These assessments were based on clinical interview and behavioral observation in accordance with routine emergency psychiatric practice, rather than on a structured research diagnostic interview. Whenever possible, this judgment was informed not only by direct interview and observation, but also by information available in the PESA record and other sources, such as earlier crisis assessments, prior information from the two largest regional mental health services, and collateral history. Although prior information from mental health institutions could be used during the acute assessment when available, the study did not have permission to retrospectively extract or link additional diagnostic information from psychiatric treatment records outside PESA and Arkin Mental Health. The assessments were conducted within a well-established psychiatric emergency service by a relatively fixed group of psychiatrists and psychiatry residents under supervision, working within shared clinical routines and regular case discussions aimed at promoting consistency in the assessment of acute presentations. No separate inter-rater reliability protocol was implemented.

Given the acute setting and the often limited opportunity for comprehensive diagnostic assessment (e.g., due to limited cooperation, intoxication, environmental circumstances or time constraints), diagnoses were grouped pragmatically into four broad categories reflecting the primary clinical presentation at the time of evaluation. Based on the classification given during the evaluation, participants were categorized into four diagnostic groups: (1) psychotic disorders, including schizophrenia, drug-induced psychosis, isolated delusional disorder, catatonia, and schizoaffective disorder; (2) substance-related disorders, encompassing drug abuse/addiction, alcohol abuse/addiction, and psycho-organic syndrome; (3) other psychiatric disorders, including manic episodes, depression, and personality disorders; (4) no primary psychiatric disorder, including cases with adjustment disorder, psychosocial problems, or situations in which no clear psychiatric disorder could be established during the assessment. Because inclusion in the program was based on police referral due to suspected mental health crisis, rather than a confirmed diagnosis, individuals for whom no psychiatric or substance use diagnosis was established during the evaluation were retained as a separate category in the analyses. In cases of comorbidity, classification was based on the clinician’s judgment of the primary disorder contributing to the acute presentation and legal decision-making at that time. Substance-induced psychosis was categorized within psychotic disorders because the presence of acute psychotic symptoms—rather than substance use per se—was considered the primary driver of clinical risk assessment and diversion decisions. Further, recommended intervention at evaluation was registered. The diagnostic categories are displayed in Table 1.

**Table 1:** Primary diagnostic categories of psychiatric evaluations at the police station

Primary diagnostic category	Examples of clinical presentations
Psychotic disorder (*N* = 109)	Schizophrenia, drug induced psychosis, isolated delusional disorder, catatonia, schizoaffective disorder
Drug or alcohol abuse and/or addiction (*N* = 58)	Drug abuse and/or addiction, alcohol abuse and/or addiction, psychoorganic syndrome
Other psychiatric disorders (*N* = 78)	Includes depression, manic episode, personality disorders
No primary psychiatric disorder (*N* = 47)	Adjustment disorder, psychosocial problems, no primary psychiatric disorder

For the legal data, we recorded the primary offence for which the individual was suspected at the time of arrest. For analytic purposes, offences were grouped into three broad categories reflecting behavioural domains rather than strict penal code classifications: Crimes were classified as (1) theft-related offences; (2) public disturbance/disruption, including arson, possession of weapons, non-compliance with police orders, and damage to public property; (3) interpersonal offences, defined as offences directed against another person, ranging from harassment and trespassing to physical assault. When multiple offences were recorded, cases were classified according to the most serious interpersonal component. The grouping was intended to reflect behavioural domains relevant to risk assessment and diversion decisions rather than formal penal code distinctions. It should be noted that the more severe crimes (e.g. murder and rape) were under-represented as suspected crimes. In those cases, prosecutors tended to be less likely to ask for immediate psychiatric evaluation, because these PSORPAs would be incarcerated for long periods. Furthermore, we also recorded the number of rearrests during a one-year follow-up after the index assessment. Rearrest was defined as any new police arrest for a suspected offence recorded by the Amsterdam police during the one-year follow-up period.

### Statistical analysis

The study population was stratified into four groups according to their diagnostic categories (Table 1). Initial analyses were conducted to compare baseline characteristics, clinical and judicial outcomes across these groups to identify any significant differences.

To assess the impact of the primary diagnostic category on the frequency of rearrests, a series of negative binomial regression analyses were performed. Negative binomial regression was chosen due to the count nature of the outcome variable (number of rearrests) and the presence of overdispersion. The analysis proceeded in three stages:

Identification of covariates: preliminary negative binomial regression models were fitted individually for each potential predictor variable, including both patient-related factors (e.g., demographic characteristics at baseline and the outcomes of the evaluation) and the number of rearrests as the outcome variable using GENLIN procedure in SPSS. This step aimed to identify relevant predictor variables that demonstrated a significant association with the outcome.

Primary model fitting: a basic negative binomial regression model was then constructed with the number of rearrests as the outcome variable and the main diagnostic category as the predictor variable. This model served to estimate the unadjusted effect of diagnostic category on the likelihood of rearrests.

Multivariate model development: finally, a comprehensive multivariate negative binomial regression model was fitted, incorporating the main diagnostic category as the primary predictor and the other significant predictor variables identified in the first step as covariates.

Correlations between the main independent variables were examined before interpretation of the multivariable model. These were small to modest and did not suggest strong pairwise overlap between predictors.

Model fit was assessed using Likelihood Ratio Chi-Square, and results are reported as incidence rate ratios (IRRs) with 95% confidence intervals (CIs) to quantify the strength and direction of associations. Multivariable analyses were conducted using complete-case analysis. *P* values < 0.05 were considered statistically significant.

## Results

### Composition of cohort

**Fig. 1:**
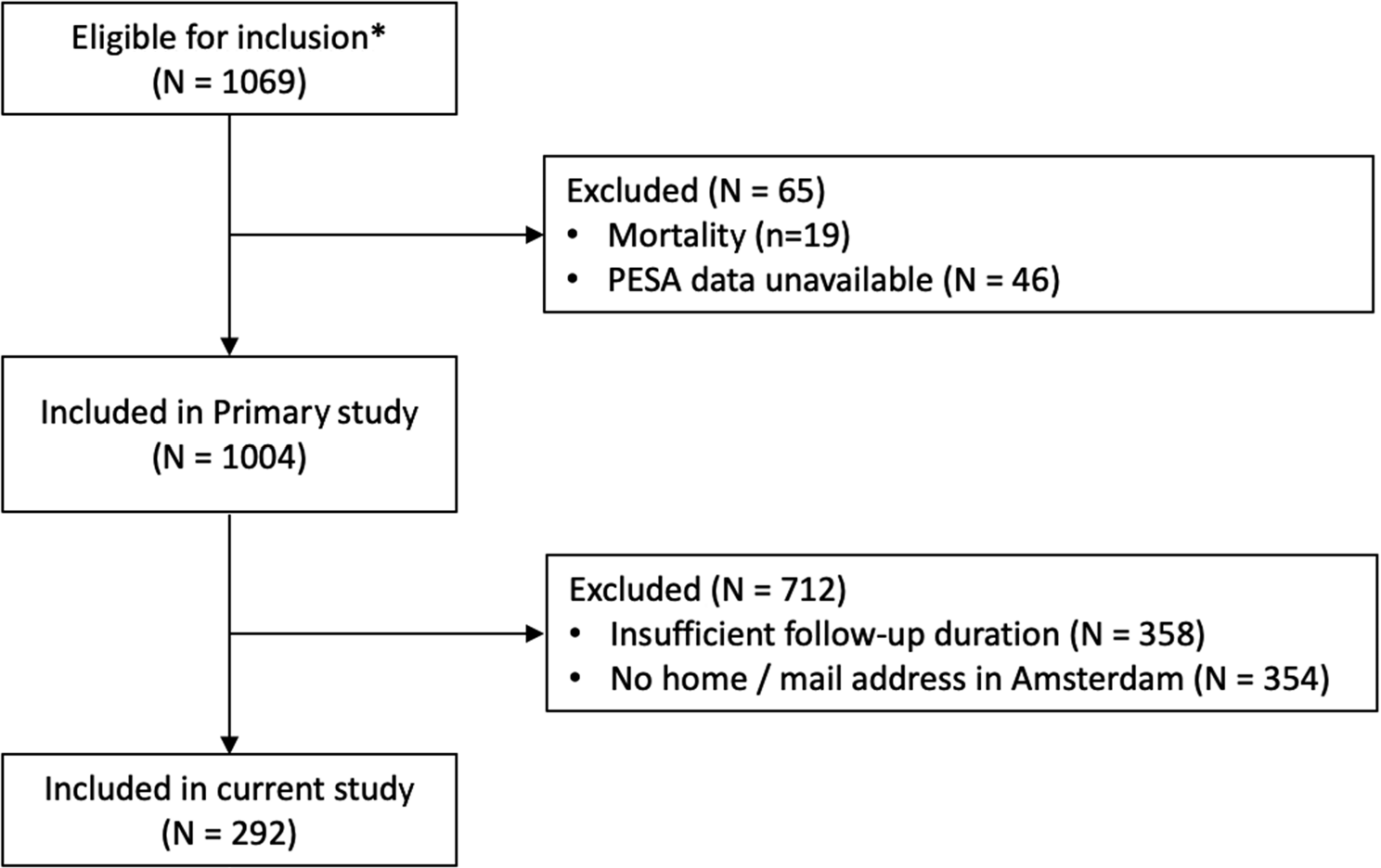
Patient inclusion

In the original cohort, 1069 PSORPA received a psychiatric assessment as part of the post-booking diversion program. After exclusions because of mortality (*n* = 19) and insufficient available data (*n* = 46), 1004 PSORPA were enrolled. After exclusion of individuals with insufficient follow-up duration (*n* = 354) or without a registered residential or postal address in Amsterdam (*n* = 358), a total of 292 subjects were included in the present study (Fig. 1). These individuals were reasonably assumed to have remained within the region during the one-year follow-up period, allowing for the availability of sufficient follow-up data. Compared with the excluded group, the included sample comprised a higher proportion of females (24% vs. 10%) and was, on average, older (mean age 40 vs. 35 years). Participants in the included group were also more likely to hold Dutch nationality and nearly 40% received welfare benefits. Importantly, no significant differences were observed between Amsterdam-based study cohort and the excluded group with respect to psychiatric diagnoses or treatment recommendations resulting from the psychiatric consultation. Of 39 (13%) subjects, the suspected crime at the time of inclusion was unknown. Additionally, the decision on criminal charges at the time of inclusion was unknown of 88 (30%) subjects.

Psychiatric evaluations revealed that patients with psychotic disorders formed the largest group of our population, followed by the categories other psychiatric disorders, drug or alcohol related problems and no disorder. Comparative analysis was conducted to explore disparities in socio-demographic and judicial characteristics across the four diagnostic categories. The four groups did not differ with regards to sex, living situation, nationality and nature of suspected crime at inclusion (Table 2). A significant difference in age was observed among the groups (Chi^2^*p* 0.023).

### Judicial and clinical outcomes by diagnostic category

Suspected crimes, clinical and judicial outcomes were compared among the four diagnostic categories. The prosecutor’s decision on criminal charges after consultation by the psychiatrist differed among the four groups. The psychotic disorder group had a larger percentage of dismissal of penalty than the alcohol and drug related disorder group (44% vs. 11%, Chi^2^ p 0.015).

Also, clinical decisions after the evaluation differed significantly among the groups (Chi^2^*p* < .001); participants with a psychotic disorder were admitted to a psychiatric clinic more often than those from the other groups; in fact, almost all hospitalized patients had a psychotic disorder. Participants with no psychiatric disorders only rarely received psychiatric treatment and none were hospitalized.

### Repeat police arrests

Within one year of program entry, 123 out of 292 participants (42%) experienced at least one rearrest (Table 2). The distribution of rearrests was highly skewed and zero-inflated; therefore, the outcome was categorized into 0, 1, and > 1 rearrests to distinguish between no, single, and repeated reoffending. Although rearrests were categorized descriptively into 0, 1, and > 1 events, regression analyses were performed using the original count variable. Diagnostic categories did not differ significantly in the number of rearrests (0, 1, > 1; χ² *p* = .144).

To identify factors associated with rearrests, to be included as covariates in a multivariate model, bivariate negative binomial regression analyses were conducted with the number of rearrests as the outcome variable and various patient-related factors as predictor variables (Table 3). Results are presented as incidence rate ratios (IRRs) with 95% confidence intervals (CIs).

Seven of the ten examined variables were significantly associated with the number of rearrests in the bivariate analyses (Table 3). First, socio-demographic variables showed that having a registered address in Amsterdam was associated with a lower rate of repeat arrests compared with homelessness (IRR = 0.37, 95% CI 0.24–0.58, *p* < .001), whereas being on welfare was associated with a higher rate of repeat arrests (IRR = 1.58, 95% CI 1.15–2.16, *p* = .004). Second, regarding clinical characteristics, individuals with a drug- or alcohol-related disorder had a higher rate of repeat arrests compared with those without a primary psychiatric diagnosis (IRR = 1.92, 95% CI 1.17–3.15, *p* = .010). Third, prior criminal justice involvement was strongly associated with rearrest: each additional police arrest in the year preceding inclusion increased the rate of repeat arrests (IRR = 1.23, 95% CI 1.14–1.33, *p* < .001). Fourth, regarding the suspected crime at the time of inclusion, interpersonal offences were associated with fewer rearrests compared with theft (IRR = 0.53, 95% CI 0.35–0.80, *p* = .003). For this analysis, data from 253 participants were available, as information on suspected crime was missing for 39 individuals; a representativeness test showed no significant differences between these groups. Fifth, in the category of judicial decisions following consultation, receiving a punishment order including a fine was associated with fewer rearrests compared with dismissal of charges or subpoena or arraignment (IRR = 0.35, 95% CI 0.17–0.75, *p* = .006). Finally, with regard to clinical intervention following psychiatric evaluation, admission to a psychiatric clinic was associated with fewer repeat arrests compared with no planned psychiatric treatment (IRR = 0.35, 95% CI 0.19–0.65, *p* = .001). Sex, age, nationality, and outpatient treatment after psychiatric evaluation were not significantly associated with repeat arrests in the bivariate analyses.

**Table 2:** Socio-demographic characteristics, legal parameters and outcome of psychiatric evaluation by diagnostic category (*N* = 292)

			Primary diagnostic category					
		Total *N* = 292	Psychotic disorder *N* = 109	Drug or alcohol related *N* = 58	Other psychiatric disorders *N* = 78	No disorder *N* = 47	Chi2	*P*
Sex	M (%*)	222(76)	85 (78)	49(85)	54(69)	34(72)	4.831	0.185
Age	Younger than 30 (%)	73 (25)	26 (24)	12 (21)	19 (24)	16 (34)	14.617	0.023
	30–40 (%)	92(32)	33 (30)	29 (50)	21 (27)	9 (19)		
	Older than 40 (%)	127 (43)	50 (46)	17 (29)	38 (49)	22 (47)		
Living situation	Registered address in Amsterdam (%)	258 (88)	101 (93)	50 (86)	68 (87)	39 (83)	6.366	0.384
	Homeless in Amsterdam(%)	34 (12)	8 (7)	8 (14)	10 (13)	8 (17)		
On welfare	No (%)	176 (60)	65 (60)	35 (60)	48 (62)	28 (60)	0.0181	0.994
	Yes (%)	116 (40)	44 (40)	23 (40)	30 (39)	19 (40)		
Nationality	Netherlands (including Dutch Antilles) (%)	226 (77)	88 (81)	46 (79)	57 (73)	34 (72)	3.663	0.722
	Europe (EU and other) (%)	26 (9)	8 (7)	4 (7)	10 (13)	4 (9)		
	Other countries and unknown (%)	41 (14)	13 (12)	8 (14)	11 (14)	9 (19)		
Police arrests one year before inclusion	0–1 (%)	192 (66)	84 (77)	27 (47)	49 (63)	32 (68)	19.160	0.004
	2–3 (%)	67 (23)	20 (18)	18 (31)	19 (24)	10 (21)		
	›3 (%)	33 (11)	5 (5)	13 (22)	10 (13)	5 (11)		
Suspected crime during inclusion*	Theft (%)	65 (26)	23 24	12 25	19 28	11 (27)	1.276	0.973
	Interpersonal offences (%)	111 (44)	42 (44)	20 (41)	30 (45)	19 (46)		
	Public disturbance and disruption (%)	76 (30)	30 (32)	17 (35)	18 (27)	11 (27)		
Intervention after psychiatric evaluation	Outpatient treatment (%)	102 (35)	39 (36)	21 (36)	30 (39)	12 (26)	60.601	< 0.001
	Admission (%)	34 (12)	32 (29)	1 (2)	1 (1)	0 (0)		
	No planned psychiatric treatment (%)	155 (53)	38 (35)	36 (62)	46 (60)	35 (75)		
Decision on criminal charges**	subpoena or arraignment (%)	125 (61)	37 (48)	29 (76)	38 (66)	21 (68)	15.850	0.015
	punishment order including a fine (%)	19 (9)	6 (8)	5 (13)	5 (9)	3 (10)		
	dismissal of a penalty (%)	60 (29)	34 (44)	4 (11)	15 (26)	7 (23)		
Number of arrests following crimes 1 year after inclusion	0 (%)	169 (58)	70 (64)	28 (48)	41 (53)	30 (64)	9.575	0.144
	1 (%)	50 (17)	19 (17)	8 (14)	16 (21)	7 (15)		
	> 1 (%)	73 (25)	20 (18)	22 (38)	21 (27)	10 (21)		

* % within Primary diagnostic category

** Of 39 (13%) subjects, the suspected crime during inclusion is unknown

*** Of 88 (30%) subjects, the decision on criminal charges during inclusion is unknown

**Table 3:** Bivariate negative binomial regression (GENLIN): Socio-demographic, clinical and legal predictors of repeat police arrests (*N* = 292)

		Parameter Estimates				Omnibus test		
		IRR	95% CI		Wald *P*	Likelihood Ratio Chi2	df	*P*
			Lower	Upper				
Sex (*n* = 292)	male	0.72	0.95	2.03	0.21	2.906	1	0.09
	female	1.00	1.00	1.00				
Age (linear) (*n* = 292)		1.01	1.00	1.02	0.09	2.973	1	0.09
Living situation (*n* = 292)	Registered address in Amsterdam	0.37	0.24	0.58	< 0.001	19.782	1	< 0.001
	Homeless in Amsterdam	1.00	1.00	1.00				
Nationality (*n* = 292)	Netherland (includes Dutch Antilles)	1.11	0.70	1.77	0.65	4.755	2	0.09
	Europe (EU and other)	1.87	0.99	3.54	0.054			
	Other countries	1.00	1.00	1.00				
On welfare (*n* = 292)	Yes	1.58	1.15	2.16	0.004	8.155	1	0.004
	No	1.00	1.00	1.00				
Primary diagnostic category (*n* = 292)	Psychotic disorder	0.67	0.41	1.08	0.10	25.866	3	< 0.001
	Drug or alcohol related	1.92	1.17	3.15	0.01			
	Other psychiatric disorders	0.95	0.58	1.56	0.85			
	No disorder	1.00	1.00	1.00				
Number of police arrests one year before inclusion (*n* = 292)		1.23	1.14	1.33	< 0.001	42.348	1	< 0.001
Suspected crime during inclusion (*n* = 253*)	Public disturbance and disruption	0.99	0.65	1.52	0.97	12.840	2	0.002
	Interpersonal offences	0.53	0.35	0.80	0.003			
	Theft	1.00	1.00	1.00				
Decision on criminal charges after inclusion (*n* = 204**)	punishment order including a fine	0.35	0.17	0.75	0.006	21.182	2	< 0.001
	subpoena or arraignment	1.11	0.58	2.10	0.76			
	dismissal of a penalty	1.00	1.00	1.00				
Intervention after psychiatric evaluation (*n* = 292)	Outpatient treatment	0.81	0.58	1.13	0.21	12.178	2	0.002
	Admission	0.35	0.19	0.65	0.001			
	No planned psychiatric treatment	1.00	1.00	1.00				

Table 3 presents bivariate associations based on the largest available sample for each predictor; sample size therefore varies across predictors due to missing data

* Of 39 of 292 (13%) subjects, the suspected crime during inclusion is unknown

** Of 88 of 292 (30%) subjects, the decision on criminal charges during inclusion is unknown

CI, confidence interval; df, degrees of freedom; IRR, incidence rate ratio

**Table 4:** Multiple regression analysis stepwise (method: GENLIN, negative binomial): Diagnostic category, socio-demographic, clinical and legal variables as independent predictors of number of repeat police arrests (*N* = 292)

		Step 1 (N = 292)				Step 2 (N = 198)			
		Parameter Estimates				Parameter Estimates			
		IRR	95% CI		Wald P	IRR	95% CI		Wald P
			Lower	Upper			Lower	Upper	
Primary diagnostic category	Psychotic disorder	0.67	0.41	1.08	0.10	1.28	0.63	2.58	0.49
	Drug or alcohol related	1.92	1.17	3.15	0.01	1.67	0.83	3.34	0.15
	Other psychiatric disorders	0.95	0.58	1.56	0.85	1.35	0.69	2.64	0.38
	No disorder	1				1			
Living situation	Homeless in Amsterdam					2.41	1.32	4.37	< 0.01
	Registered address in Amsterdam					1			
On welfare	Yes					1.54	1.01	2.36	0.045
	No					1			
Police arrests one year before inclusion (lineair)						1.16	1.07	1.27	< 0.01
Suspected crime during inclusion*	Public disturbance and disruption					0.82	0.46	1.45	0.50
	Interpersonal offences					0.71	0.42	1.18	0.19
	Theft					1			
Decision on criminal charges after inclusion**	Punishment order including a fine					2.28	0.93	5.60	0.07
	Subpoena or arraignment					2.70	1.48	4.93	< 0.01
	dismissal of a penalty					1			
Intervention after psychiatric evaluation	Outpatient treatment					1.09	0.68	1.75	0.73
	Admission					0.83	0.33	2.11	0.70
	No planned psychiatric treatment					1			
Omnibus Test		Likelihood Ratio Chi-Square	df	Sig.		Likelihood Ratio Chi-Square	df	Sig.	
		25.867	3	< 0.001		76.623	12	< 0.001	
		Change				50.756	9	< 0.001	

Multivariable model based on complete-case analysis (*N* = 198). Participants with missing data on one or more covariates were excluded from the final model. Diagnostic group composition in Step 2: psychotic disorder *n* = 76, drug- or alcohol-related disorder *n* = 38, other psychiatric disorders *n* = 55, and no disorder *n* = 29

* Of 39 (13%) subjects, the suspected crime during inclusion is unknown

** Of 88 (30%) subjects, the decision on criminal charges during inclusion is unknown

CI, confidence interval; df, degrees of freedom; IRR, incidence rate ratio

### Determinants of repeat police arrests

To evaluate the relationship between main diagnostic category and the number of rearrests, first (step 1) a negative binomial model was fitted with number of rearrests as outcome variable and main diagnostic category as predictor variable. Diagnostic category was significantly associated with recurrent arrest, with the substance use disorder group having more rearrests than the reference group (IRR 1.92, CI1.17-3.15, *P* = .01, Table 4). Next, (step 2) all significant predictors of rearrests that were identified with the bivariate analyses (i.e. living situation, welfare status, number of police arrests one year before inclusion, suspected crime during inclusion, decision on criminal charges after inclusion, intervention after psychiatric evaluation) were added to the model as covariates (Table 4). The final multivariable model was based on 198 participants with complete data on all covariates. The reduction in sample size was due to missing data on one or more of the additional variables entered in Step 2, particularly variables relating to suspected crime and decision on criminal charges. This reduced the number of observations in all diagnostic categories but did not materially alter their relative distribution in the analytic sample.

After correction, main diagnostic category was not a statistically significant predictor of rearrests anymore. The covariates that emerged as statistically significant predictors of rearrests were: receiving subpoena or arrangement after inclusion in the study (IRR 2.70, CI 1.48–4.93, *P* = .001), experiencing homelessness in Amsterdam (IRR 2.41, CI1.32-4.37, *P* = .004), being on welfare (IRR 1.54, CI1.01-2.36, *P* = .045), and number of police arrests in the year before inclusion (IRR 1.16, CI1.07-1.27, *P*<.001).

## Discussion

### Rearrest in 1 year follow up in the Amsterdam postbooking diversion program

Jail based post-booking diversion programs aim to improve mental health outcomes of individuals suspected of criminal behavior with a potential concurrent mental health crisis, and to reduce overrepresentation of persons with severe mental illness in the prison system. A post-booking program was implemented in Amsterdam, which involved (1) PSORPA s with a possible mental health crisis receiving a psychiatric evaluation in the jail cell by PESA staff, (2) subsequent consultation between the consulting PESA psychiatrist and the prosecutor’s office and (3) possible transfer of the PSORPA to PESA for treatment rather than prosecution. Here, we report the 1-year outcomes of this program in terms of rearrests and their predictors.

### Post-booking diversion and rearrest: psychiatric diagnoses and other predictors

We found that 42% of participants in the program had one or more rearrests in the following year, the remainder did not experience a rearrest. Virtually all PSORPA s who were diverted to inpatient care were those with a psychotic episode. Interestingly, after adjustment for covariates, the diagnostic classification assigned by PESA staff after the evaluation did not predict the occurrence of rearrests, and those who were admitted to a psychiatric clinic had no significantly different rate for rearrest than those not being referred for treatment. In other words: in our sample of patients who are assessed by police officers as potentially experiencing a mental health crisis, when given acute in-patient treatment, when necessary, psychotic patients do not experience rearrest more often than those without a psychiatric disorder. If indeed psychotic disorders are a risk factor for recidivism or rearrest [14], this would point to a reduction in rearrest by adequate clinical treatment. And although due to the lack of a control group the latter statement is of a speculative nature, it is in line with the available literature. For instance, a study evaluating 248 persons with co-occurring severe mental disorders and substance abuse disorders, who were arrested and booked on misdemeanor charges, did not find a difference in rearrest rates in those who were diverted (*n* = 154) when compared with those who were not (*n* = 94) [29]. And a study following 125 individuals who were diverted post-booking to mental health care services, found that those completing the program had a smaller risk of being arrested [11].

A negative binomial model showed that substance use disorder was initially associated with more rearrests, but this association was no longer significant after adjustment for other predictors. Rearrests were associated with receiving subpoena or arrangement, in comparison with dismissal or a fine. These findings should be interpreted cautiously, as attenuation in adjusted models may arise from confounding or correlated covariates rather than reflecting a specific causal pathway. The higher likelihood of rearrest following a subpoena or arraignment, compared to dismissal or a fine, could be related to the greater seriousness of these cases. Court involvement may also be influenced by factors such as stigma, increased monitoring, or the social and psychological strain of legal proceedings [30]. Although the study was not designed to identify causal predictors of rearrest, homelessness and lack of welfare support were independently associated with rearrest in the adjusted model. This supports the notion that conceptualizing mental illness as the sole cause of criminal involvement is overly simplistic and lacks utility for policy or service implications. Indeed, when examining the factors contributing to incarceration or re-incarceration, it becomes evident that social difficulties, often compounded by mental illness but not exclusively caused by it, play a crucial role [31].

These conclusions are consistent with the findings of Draine et al., who argue that the relationship between serious mental illness and social problems—such as crime, unemployment, and homelessness—is far more influenced by broader social context, particularly poverty, than by mental illness alone [32]. And in their literature review, Skeem et al. demonstrated that system solutions (including diversion programs) primarily focused on symptom management yield limited effectiveness in reducing recidivism rates [33]. On the other hand, housing programs in the absence of additional interventions are also unlikely to reduce future contact with the criminal justice system. Based on interviews with 584 participants of a multisite trial of Housing First in Canada, of whom 59% were rearrested in 2-year follow-up, Roy et al. questioned the appropriateness of laws and regulations that target survival behaviors of the homeless or practices that address psychosocial issues like substance use disorders through a punitive rather than rehabilitative approach [34].

To reduce the risk of reoffending of PSORPA s experiencing a mental health crisis, comprehensive solutions covering symptomatic control and improving social conditions, such as Assertive Community Treatment (ACT) and assisted housing programmes, may be more beneficial to the patient and society than treatment focused only on symptom reduction [35]. In the Netherlands, outreach-oriented Flexible ACT (FACT) teams and municipal “bemoeizorg” services similarly aim to integrate clinical treatment with practical support in areas such as housing, income, and social functioning [36]. Such models may offer a more appropriate framework for individuals experiencing mental health crises in combination with homelessness and repeated criminal justice contact.

Although beyond the scope of the present study, future work may consider applying path analysis to better understand the mechanisms linking behavioral health conditions, social determinants, and subsequent rearrest.

### Strengths and limitations

The study has several strengths. First, the program entails a close collaboration between the psychiatrist and prosecutor’s office, allowing for concurrent clinical and legal decision-making. Second, the project provides valuable real-world data by having recruited all patients who have been evaluated in the program over a pre-defined time period. Third, the data sharing covenant of the multiple participating partners, allowed for the formation of a comprehensive dataset including sociodemographic, clinical and judicial data.

The study also has its limitations. Most importantly, the methodology did not encompass a control group. Thus, although we were able to evaluate the outcomes of the program, we were unable to assess the effectiveness of the program compared to the situation preceding it. Selection bias is another potential limitation. Patients could only be enrolled in the program when the jail police officers suspected that they were experiencing a mental health crisis. As police officers are not extensively trained in the identification of mental health disorders, a subset of eligible participants may have gone unnoticed. At the same time, this reflects the real-world operational context of the program and may therefore be difficult to avoid in practice.

A further limitation concerns the diagnostic classifications. It should be stressed that the main diagnostic categories do not refer to diagnoses established after guideline-adherent procedures. Rather, they refer to the PSORPAs main problems, which were identified under challenging circumstances of a brief psychiatric evaluation, in a jail complex, often with an intoxicated or uncooperative person [37]. The diagnostic categories do not systematically capture psychiatric comorbidity, including co-occurring substance use disorders, and formal inter-rater reliability was not assessed. On the other hand, these classifications are derived from routine clinical practice and reflect real-time assessments guiding immediate decision-making, supporting their relevance to real-world settings. The analyses also did not adjust for time at risk, as some participants spent periods in inpatient or custodial settings during follow-up. While this may have reduced exposure to rearrest, these periods also reflect intended clinical or judicial interventions, and confinement does not preclude criminal behaviour. In addition, the analytic sample was restricted to individuals with documented ties to Amsterdam and available one-year follow-up data. This restriction was applied to ensure reliable follow-up within the same local justice and healthcare systems and to reduce the likelihood of missing outcome information due to relocation outside the region. However, this approach may limit the generalizability of the findings to the broader population of all PSORPAs referred for psychiatric evaluation. Moreover, the final multivariable model was based on complete-case analysis, meaning that participants with missing data on one or more covariates were excluded. This further reduced the sample size, may have lowered statistical power, and limits the generalizability of the adjusted findings.

Finally, several limitations relate to the analytic strategy. The multivariable model does not identify causal or definitive predictors but rather estimates associations that are conditional on the covariates included in the model. Decisions about which factors to include or adjust for are inherently complex, and multiple statistical comparisons increase the likelihood of chance findings, while results may also be susceptible to post-hoc interpretation, all of which can make conclusions less robust [38]. Variable selection was informed by univariate associations prior to multivariable modelling. As a consequence, predictors with non-significant univariate associations may nonetheless have been relevant in the multivariable context after adjustment for other covariates. While this approach has recognised limitations, it is one of several commonly used strategies in exploratory analyses. Given the exploratory aims of the study and the limited feasibility of assembling comparable datasets, this approach was considered reasonable in the present context; findings should therefore be interpreted as hypothesis-generating.

## Conclusion

The Amsterdam jail-based post-booking diversion program demonstrates how collaboration between mental health services, police and prosecutors may help address the intersection of mental health crises and the criminal justice system.

While psychotic disorders were strongly associated with hospitalization and dismissal of charges, psychiatric diagnosis itself was not associated with rearrest within one year.

In contrast, social vulnerability—particularly homelessness and lack of welfare support—was independently associated with rearrest.

The high rate of rearrests among individuals experiencing homelessness is consistent with other research suggesting that diversion programs may benefit from adopting a broader scope, integrating social support and housing interventions alongside clinical care.

## Data Availability

Although data property rights are owned by the Arkin Research Department and Actiecentrum Veiligheid en Zorg, City of Amsterdam, researchers JZ and JD held intellectual rights on data storage and use, only to the extent necessary for the abovementioned scientific research purposes, until the study was approved for publication and so are not publicly available.

## Abbreviations

PSORPA: Person suspected of an offence and referred for psychiatric assessment
PESA: Psychiatric Emergency Service Amsterdam
ACT: Assertive Community Treatment
